# Frequency of Local Allergic Asthma in Bronchial Asthma Patients: Role of Conjunctival Challenge and Neutrophil Histamine Release

**DOI:** 10.3390/cells15151381

**Published:** 2026-07-30

**Authors:** Adrián López-Postigo, Antonio Vega-Rioja, Claudia Rodríguez-Neira, Javier Monteseirín

**Affiliations:** 1 Laboratorio de Inmunología y Alergia-FISEVI, UGC de Alergología, Hospital Universitario Virgen Macarena, 41009 Sevilla, Spain; adrianlopezpostigo@gmail.com (A.L.-P.); macrofago@us.es (A.V.-R.); claudiarodriguezneira@juntadeandalucia.es (C.R.-N.); 2 Departamento de Medicina, Facultad de Medicina, Universidad de Sevilla, 41009 Sevilla, Spain; 3 Hospital Quirón Sagrado Corazón, 41013 Sevilla, Spain; 4 Hospital Quirón Infanta Luisa, 41010 Sevilla, Spain

**Keywords:** co-allergy, sensitized, local allergic asthma, asthma, allergen provocation test, histamine release, IgE, Fc receptors, allergic response, neutrophils

## Abstract

Background: There is sufficient evidence to demonstrate the importance of local allergies in the various clinical conditions encompassed by allergic pathology. We sought to examine the frequency of Local Allergic Asthma (LAA) and to investigate whether neutrophils from patients with LAA were able to release histamine compared to neutrophils from patients with asthma diagnosed by skin prick test (SPT) and specific serum IgE (sIgE). Methods: Sixty-eight patients with bronchial asthma and 15 healthy subjects were selected. All underwent SPT, and sIgE levels were determined. An allergen-specific conjunctival challenge (ASCC) and the allergen provocation test (APT) were carried out. Neutrophils were isolated, and their potential for histamine release was determined along with their influence on IgE receptors. Results: Eighteen patients had a positive ASCC and a negative SPT and sIgE to certain allergens (52.9%), coexisting in 16 patients who were SPT and sIgE-positive to other allergens (CO-LOCAL ALLERGY); these were designated LOCAL ASTHMA A (LAA). Another group of 16 patients (47.1%) only had a positive ASCC (SENSITIZED) and were designated LOCAL ASTHMA B (LAB). The total number of patients who presented a local type of asthma was 34 (50%) (TOTAL LOCAL ASTHMA (TLA)). Neutrophils isolated from patients with local asthma were capable of releasing histamine, as were neutrophils from patients diagnosed by SPT and sIgE. Conclusions: We have differentiated the clinical groups that may present LAA and have demonstrated how the neutrophils of these patients were capable of releasing histamine if stimulated with the allergen responsible for the clinical symptoms, with this release being IgE-dependent.

## 1. Introduction

Asthma is a chronic inflammatory disease that affects more than 300 million people worldwide and is currently the most common chronic respiratory condition [1,2]. Despite the countless efforts currently dedicated to understanding etiology, pathophysiology, diagnosis, phenotypes, and endotypes, it is still not fully and definitively understood.

Several phenotypes have been described, including the T2 or mainly allergic and eosinophilic type, non-T2 or non-allergic, and neutrophilic or mixed granulocytic (mixed T2, T1/T17). However, this classification is inaccurate, since studies have shown that several external factors can intervene in the temporal and causal development of the inflammatory process, making asthma very complex. The presence of neutrophils has been observed in both T2 and non-T2 subgroups despite treatment with corticosteroids, and we have verified how neutrophils can be stimulated by an IgE-dependent mechanism [3,4,5].

Local Allergy (LA) is diagnosed when patients present the typical symptoms of a particular condition (rhinitis, asthma, etc.), but the standard clinical tests practiced for the diagnosis of these processes, e.g., the SPT and sIgE, produce negative results. To demonstrate that the condition has an atopic genesis, we must use an APT. Asthma is a chronic respiratory disease with very complex characteristics in which many factors intervene that can determine a very specific clinical picture that differentiates it from other clinical types. LAA is a form of asthma that presents typical clinical symptoms such as cough, wheezing, dyspnea, chest pain, bronchial hyperreactivity with inflammation, but without usual allergic markers such as positive SPT and sIgE, requiring a positive APT for diagnosis. This study demonstrates that a potentially significant percentage of cases present with symptoms compatible with LA and can go unnoticed if appropriate diagnostic methods are not applied, since they are undetectable using conventional allergy tests [6,7,8,9,10,11].

**Figure 1:**
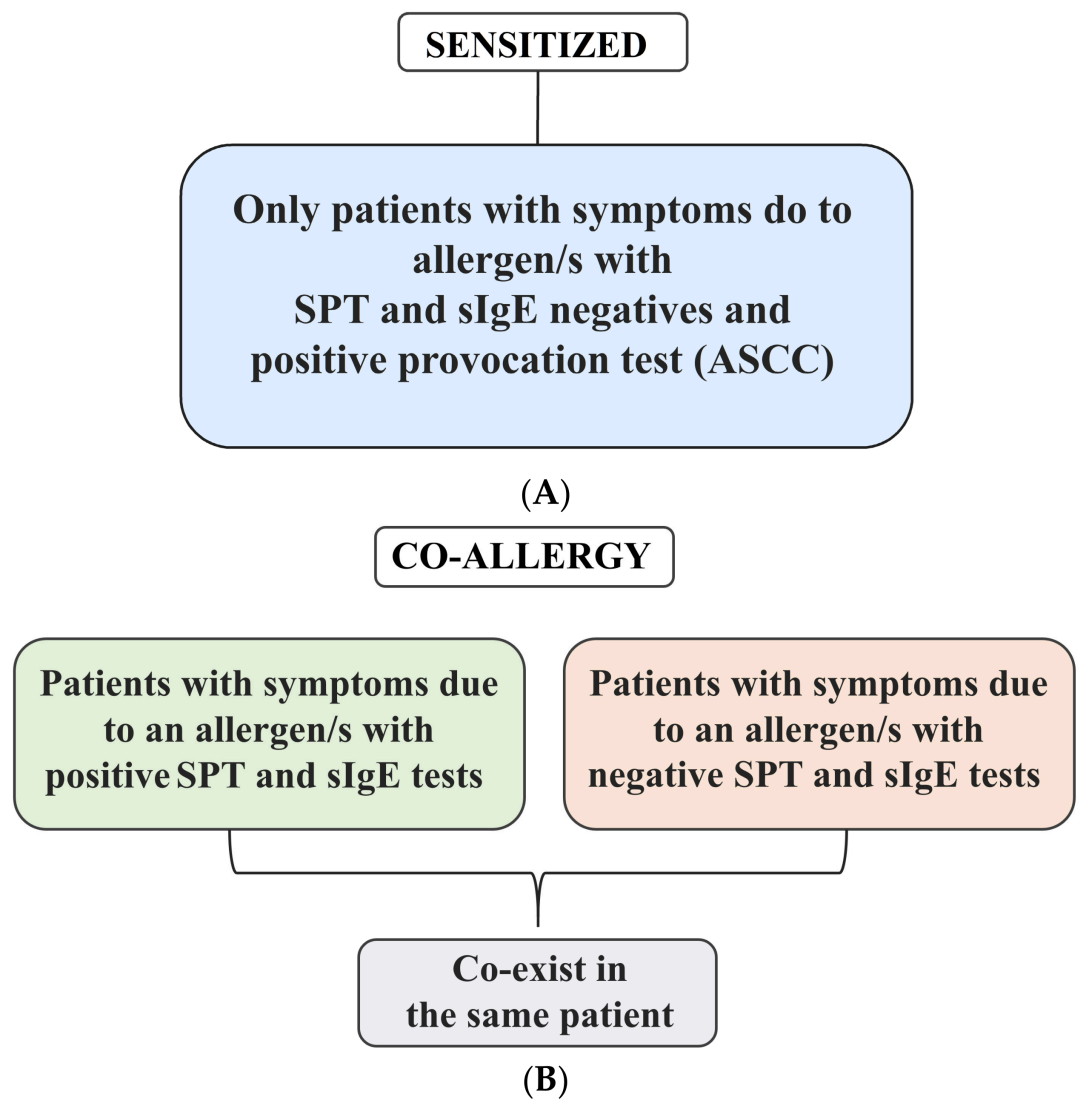
Local Allergy subtype scheme. Processes that are apparently not allergic, i.e., do not present positive skin test (SPT) and serum-specific IgE (sIgE), but nevertheless present a positive allergen provocation test (+APT = +ASCC). This has been defined as “local allergy” (Sensitized) (**A**). Allergens that can only be detected by a positive ASCC can coexist in the same person with other allergens that can be detected by SPT and sIgE (for example, the same person may be sensitive to grasses and not be detected by SPT or sIgE, and be sensitive to mites, which can be detected by SPT and sIgE) (Co-Allergy) (**B**).

In allergic processes, cells are first sensitized, and the clinical picture subsequently develops. LA involves processes that are apparently not allergic, i.e., they do not present positive SPT and sIgE to an allergen but nevertheless present a positive APT [6,7,8,9,10] (Figure 1A). It is also possible that there are allergens that can only be detected by a positive APT and that can coexist in the same person with other allergens that can be detected by SPT and sIgE. For example, the same patient can be sensitive to grasses (symptomatic in spring in Spain) and not be detected by SPT or sIgE and be sensitive to mites (symptomatic in autumn in Spain), which can be detected by SPT and sIgE. This clinical form is called Co-Local Allergy (Figure 1B).

The main objective of this study is to determine the frequency of Total Local Asthma (TLA) in subjects with bronchial asthma and the role that neutrophils may play in these patients.

## 2. Materials and Methods

### 2.1. Patients and Controls

We analyzed 34 patients diagnosed with bronchial asthma, of which *n* = 16, 47.1%, had positive SPT (ITAI Pharma, Madrid, Spain) and sIgE (detection limit of 0.35 IU/mL) (HYTEC 288; Hycor Biomedical-IZASA, Barcelona, Spain) to either house dust mites, pollens, or molds, and who also had positive ASCC to the same allergens. In the same group of 34 patients, the rest of them, *n* = 18 (52.9%), had negative SPT and sIgE to certain allergens but nevertheless had positive ASCC (LETI Pharma, Madrid, Spain). This group is called CO-ALLERGY. This sensitization would not have been evident if we had not performed an APT. We also studied 34 patients who presented symptoms of bronchial asthma, all of whom (*n* = 34) had negative SPT and sIgE; 16 (47.1%) had positive ASCC, while the rest were ASCC-negative. This group is called SENSITIZED. Finally, we included a control group of healthy adult volunteer subjects (*n* = 15) (Table 1). We randomly selected the patient population over a period of 10 years. The patients were diagnosed as asthmatic according to the European Respiratory Society Guidelines [12].

For 8 h before cell culture, patients were not allowed to take inhaled corticosteroids, short-acting and long-acting beta 2 agonists, short-and long-acting muscarinic antagonists, or montelukast. No patient was allowed to take oral corticosteroids in the week prior to cell culture. Healthy subjects did not have any allergies and had negative results for SPT (ITAI Pharma), sIgE (HYTEC 288), and ASCC (LETI Pharma). Neither patients nor healthy subjects suffered from an infectious process in the month prior to blood sampling for cell culture. No patient presented any comorbidity such as gastroesophageal reflux disease, hypertension, obstructive sleep apnea, hormonal disorders, psychiatric disorders, obesity and diabetes.

The investigations were conducted in accordance with the principles outlined in the Declaration of Helsinki (1975, revised in 2013). Each participant gave informed consent, and the Hospital (Virgen Macarena) Ethics Committee approved the study (Ref: C.I. 1772. 1999).

### 2.2. Materials

Anti-human IgE antibody (Ab) was purchased from Caltag Laboratories (Burlingame, CA, USA). Mouse and goat IgG Ab were purchased from Sigma Chemical Co. (Madrid, Spain). Mouse anti-CD203c was from IZASA (Barcelona, Spain). Mouse anti-FcεRI (α-chain) was obtained from eBioscience (San Diego, CA, USA), mouse anti-FcεRII (CD23) was from Backman-Coulter-IZASA (Barcelona, Spain), and mouse anti-galectin-3/Mac-2 was purchased from Abcam (Cambridge, UK). Lipopolysaccharide (LPS) from Escherichia coli (L2630) was from Sigma-Aldrich (Madrid, Spain). The allergens were commercially available antigen extracts, including D_1_ (*Dermatophagoides pteronyssinus*), D_2_ (*Dermatophagoides farinae*), G_3_ (*Dactylis glomerata*), G_5_ (*Lolium perenne*), T_9_ (*Olea europea*), M_6_ (*Alternaria alternata*), and W_6_ (*Artemisia vulgaris*). They were purchased from ITAI Pharma (Alcobendas, Madrid, Spain). All culture reagents used in this work (including allergens) had endotoxin levels of ≤0.01 ng/mL, as verified by the Coatest Limulus lysate assay (Chromogenix, Mölndal, Sweden). None of the reagents affected the viability of the cells at the concentrations used in this work, as confirmed by the trypan blue dye exclusion test.

### 2.3. Cell Isolation and Culture

Neutrophils were isolated and purified as previously described [13]. These highly purified neutrophils were then separated and further purified using a magnetic cell sorting system (MACS) method. The neutrophil concentration at the end of purification was >99%. Basophil contamination was excluded using anti-203c Ab staining. Although CD203c is upregulated upon basophil activation, its basal expression allows detection of resting basophils; therefore, major basophil contamination is unlikely. The cells (5 × 10^6^ cells/300 µL) were cultured in RPMI 1640 under standard conditions, with allergen concentrations as previously indicated [5].

### 2.4. Dissociation of Neutrophil-Bound Igs

The dissociation or elution of IgE molecules from the surface of neutrophils was carried out as previously described [5,14,15]. IgE molecules were dissociated from the cell-surface of neutrophils as described previously. Briefly, after isolation, neutrophils (1 × 10^8^) were resuspended in 1 mL acetate buffer (50 mM sodium acetate (pH 4), 85 mM NaCl, 5 mM KCl, supplemented with 0.03% human serum albumin) and incubated on ice for 3 min. An equal volume of gelatin veronal buffer (1.8 mM sodium barbital, 3.1 mM barbituric acid, 0.1% gelatin, 0.05 mM MgCl_2_, 141 mM NaCl, 0.15 mM CaCl_2_ (pH 7.4)) was then added to the treated cells, and the mixture was centrifuged at 500 *g* for 10 min. The igE-containing supernatant was neutralized with 1 N NaOH and used for specific IgE and IgG determination.

### 2.5. Allergen-Specific Conjunctival Challenge

The ASCC was carried out according to the method previously described by us and others, with allergens from LETI Pharma [16,17,18].

### 2.6. Analysis of Total and Released Histamine

Histamine was determined in the culture supernatant or the cellular pellets using an ELISA histamine kit from Coulter IZASA following the manufacturer’s instructions. Total cellular histamine (100% release) was obtained by lysing cells in 2% perchloric acid. The results were based on the mean of the duplicate determinations and expressed as a percentage of histamine release by dividing by total cellular histamine after subtracting for spontaneous release of unstimulated neutrophils (typically, 2–4% during the 30 min assay) [19].

### 2.7. Statistical Analysis

All statistical analyses were performed using GraphPad Prism 8.00 for Windows (GraphPad Software, San Diego, CA, USA). The sample size was calculated with a significance level of 0.05 and statistical power of 0.8. The normality of the distribution was first examined using the Shapiro–Wilk normality test before analysis of statistical significance. Data are expressed as means ± SEM. Comparisons between groups were made with one-way ANOVA. A *p*-value of less than 0.05 was considered significant. Regression analysis was performed using Pearson’s correlation coefficient.

## 3. Results

**Figure 2:**
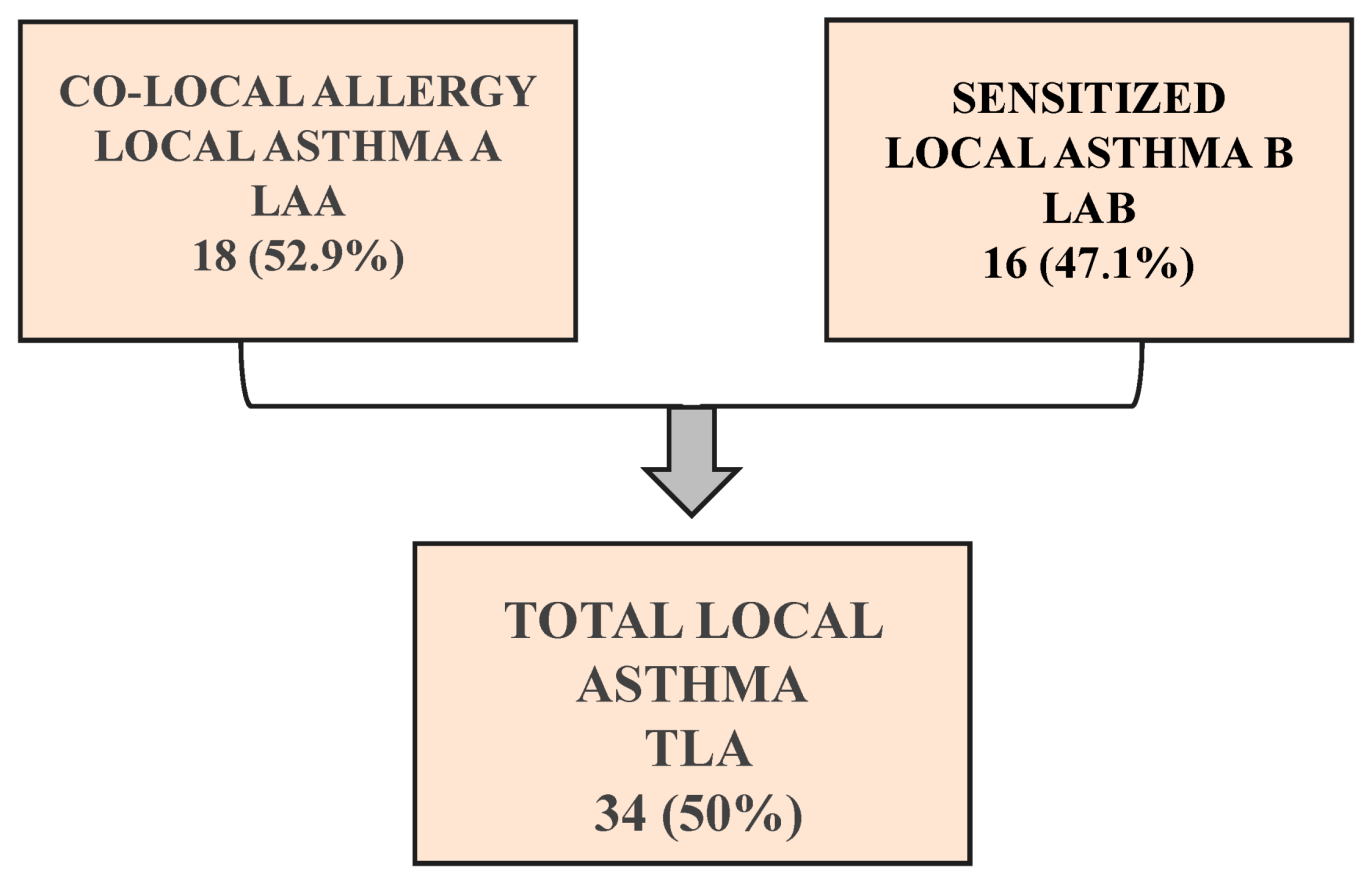
Outline of the subtypes and frequency of Local Asthma. (1) Eighteen patients (52.9%) in the Co-Local Allergy group had Local Asthma—this group will be called Local Asthma A (LAA); (2) 16 patients (47.1%) in the Sensitized group had Local Asthma—this group will be called Local Asthma B (LAB); and (3) a total of 34 patients had positive ASCC and negative SPT and sIgE, which represents 50% of all patients with asthma who may have Local Allergy—this group will be called Total Local Asthma (TLA).

First, we calculated the proportion of patients with positive results for ASCC to certain allergens, but with negative results on the SPT or sIgE tests, among the patients in the CO-ALLERGY group. Only 18 (approximately 52.9%) had positive results for ASCC (co-local allergy) and negative results on the SPT/sIgE tests; while 16 patients in the SENSITIZED group (approximately 47.1%) had negative results for SPT and IgE to the allergen panel, but positive for ASCC. In summary: (1) 18 patients (52.9%) in the Co-Local Allergy group had Local Asthma—this group will be called Local Asthma A (LAA); (2) 16 patients (47.1%) in the Sensitized group had Local Asthma—this group will be called Local Asthma B (LAB); and (3) a total of 34 patients, combining both groups, had positive ASCC and negative SPT and sIgE, which represents 50% of all patients with asthma who may have Local Allergy—this group will be called TLA (Figure 2 and Table 1).

**Figure 3:**
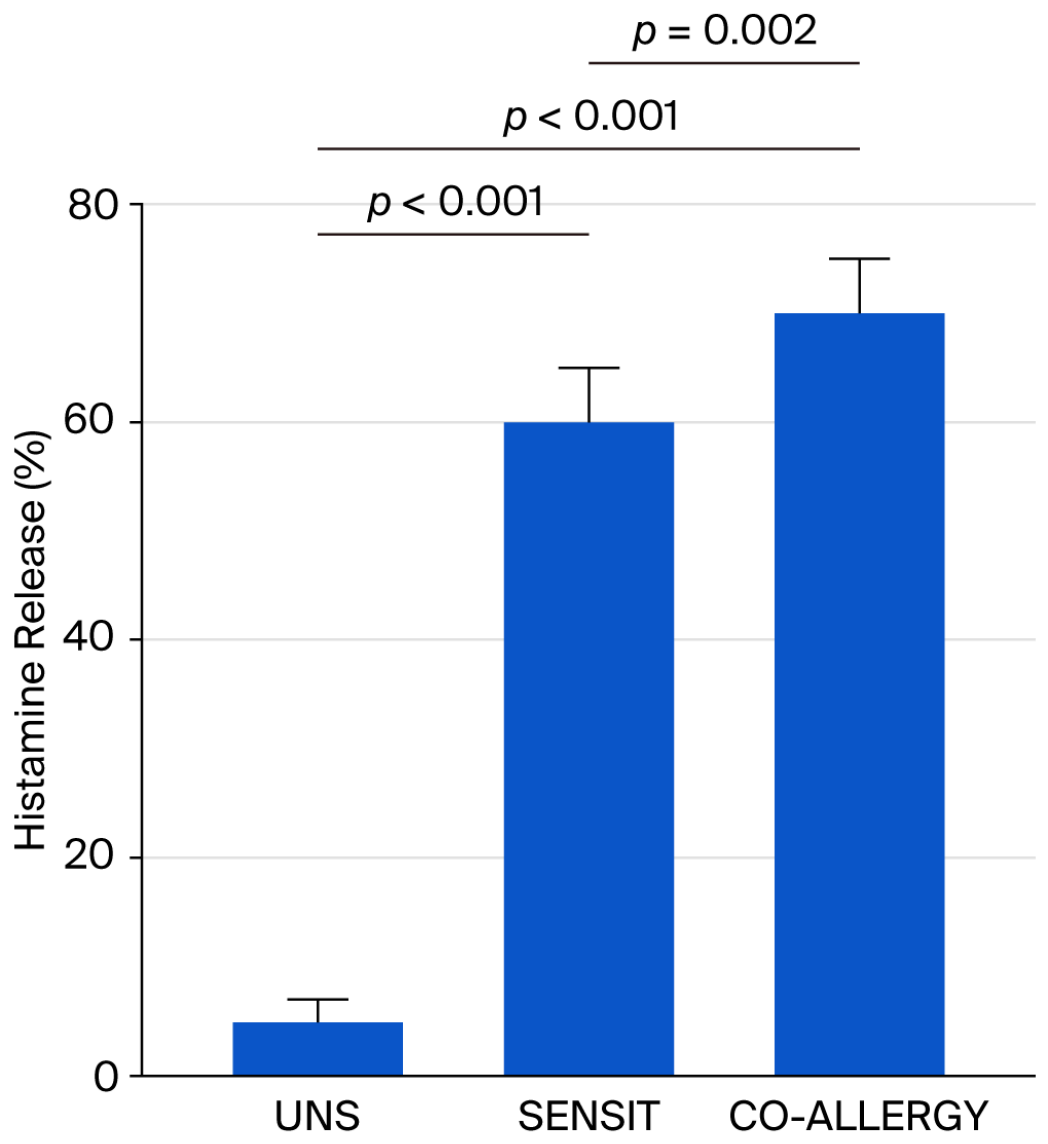
Amount of histamine released by neutrophils in patients of each LA subtype. Purified neutrophils from unstimulated patients (*n* = 68) (UNS: unstimulated), neutrophils from sensitized patients (16 patients (47.1%)) (SENSIT: Sensitized) and neutrophils from patients with Co-Allergy (*n* = 34) (CO-ALLERGY), were stimulated with an allergen to which the patient was sensitive (10 μg/mL, for 30 min), and the amount of histamine released to the culture was determined by ELISA. The amount of histamine was measured based on the mean of the duplicate determinations and expressed as a percentage of histamine release by dividing by total cellular histamine after subtracting for spontaneous release of unstimulated neutrophils. As we can see, neutrophils are capable of releasing histamine equally in patients who only show sensitivity to the allergen studied without presenting positive SPT and sIgE to that allergen. However, the amount of histamine released is statistically significantly lower (*p* = 0.02) than that released by neutrophils of patients who already demonstrate positive SPT and sIgE to other allergens. There is no histamine release if neutrophils are not stimulated (*p* < 0.001) in both types of patients.

We previously demonstrated that neutrophils were capable of releasing histamine after stimulating cells with an allergen in patients who demonstrated positive SPT and sIgE in a Neutrophil Allergen Test (NAT), but we did not know if the neutrophils of patients who had a positive ASCC and a negative SPT and sIgE were also capable of releasing histamine. As we can see, neutrophils are capable of releasing histamine in patients who only show sensitivity to the allergen studied by an ASCC without presenting positive SPT and sIgE to that allergen. However, the amount of histamine released is statistically significantly lower (*p* = 0.02) than that released by neutrophils from patients with SPT and positive sIgE to the allergen studied. There is no histamine release if neutrophils are not stimulated (*p* < 0.001) in both types of patients (Figure 3).

**Figure 4:**
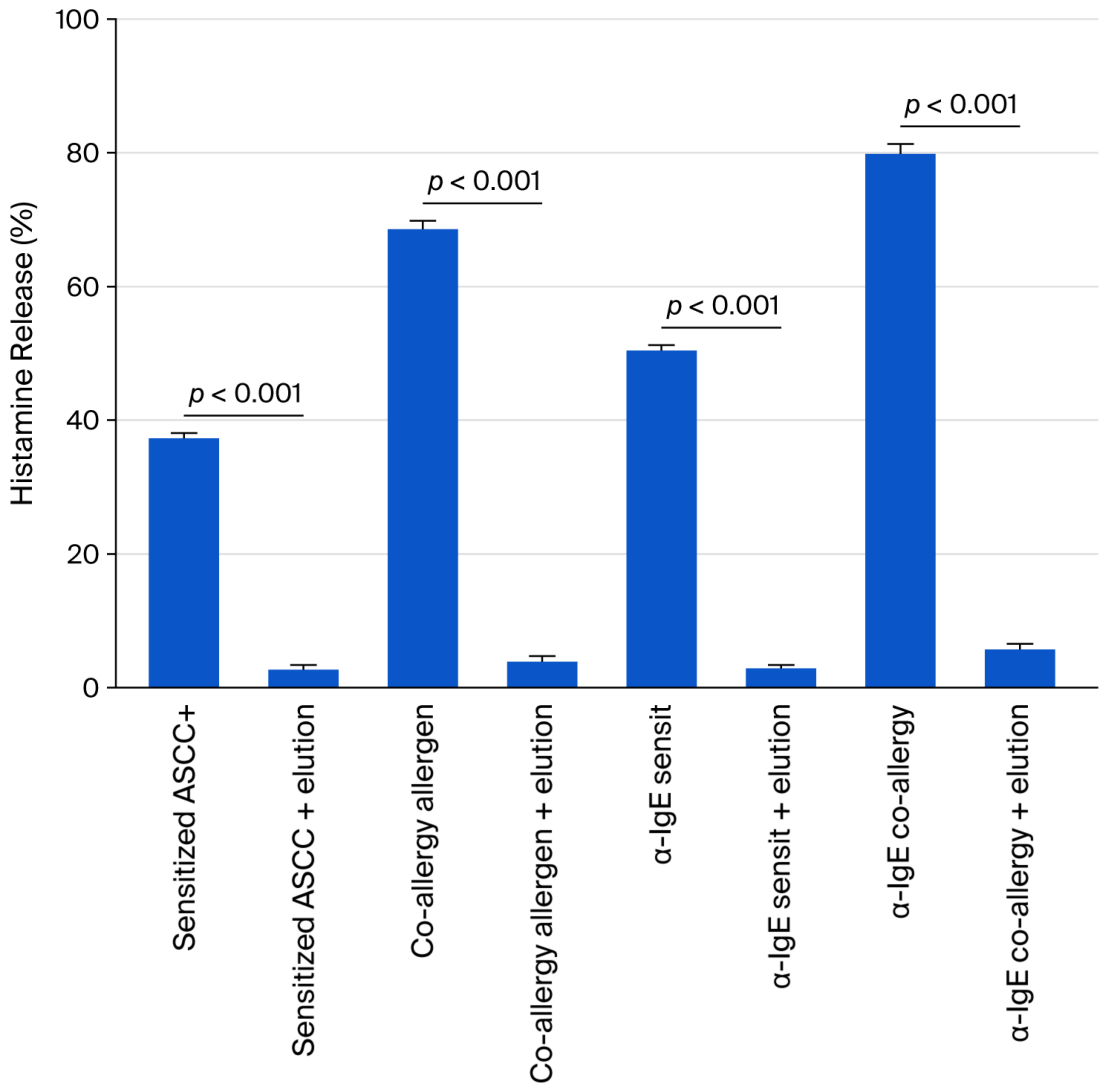
The presence of IgE on the cell surface was essential for NAT. To verify that the presence of IgE on the cell surface was essential for NAT, we eluted this immunoglobulin from the cell surface of neutrophils. Neutrophils (5 × 106 cells/300 µL) were eluted from the cell surface by a method already described. They were stimulated with an allergen to which the patient was sensitive (10 μg/mL, for 30 min), and the amount of histamine released into the culture was determined by ELISA. No histamine was released in Sensitized patients (*n* = 16) (sensitized + ASCC) or patients with Co-Allergy (*n* = 34). The amount of histamine was measured based on the mean of the duplicate determinations and expressed as a percentage of histamine release by dividing by total cellular histamine after subtracting for spontaneous release of unstimulated neutrophils.

**Figure 5:**
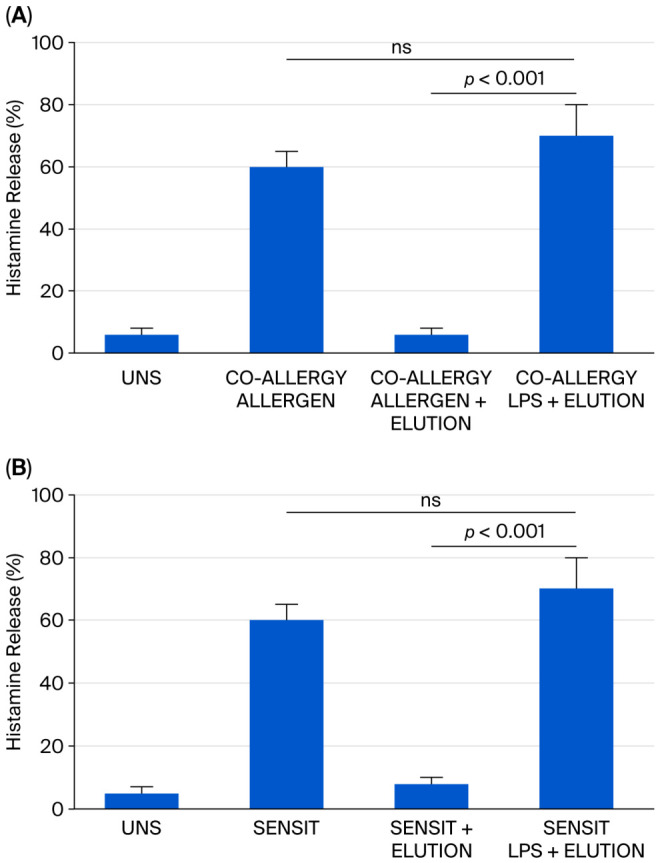
Effect on NAT of using a non-specific stimulus (**A**) and of the elution of IgE from the cell surface (**B**). If we activate neutrophils (5 × 10^6^ cells/300 µL) with an activator such as LPS (1 µg/mL, 30 min), which acts as the prototypical endotoxin because it binds the CD14/TLR4/MD2 receptor complex, and elute the IgE from the surface of neutrophils, there is no release of histamine after stimulation with the allergen to which the patient is sensitive. The same is true in subjects who present Co-Allergy (CO-ALLERGY) (*n* = 34) (**A**) and in subjects who are only sensitive (SENSIT) (**B**) (*n* = 16). In both cases, although we elute the IgE from the cell surface, a release of histamine occurs if we stimulate with LPS. The amount of histamine was measured based on the mean of the duplicate determinations and expressed as a percentage of histamine release by dividing by total cellular histamine after subtracting for spontaneous release of unstimulated neutrophils. ns = not significant.

As we can see in Figure 4, if we elute IgE from the surface of neutrophils, there is no histamine release after stimulation with the allergen to which the patient is sensitive. If we stimulate neutrophils with an anti-IgE antibody (*p* < 0.001), both in subjects who are already allergic and those who are only sensitive, there is likewise no histamine release. In both cases, although we elute IgE from the cell surface, histamine release occurs if we stimulate with LPS (*p* < 0.001) (Figure 5A,B).

**Figure 6:**
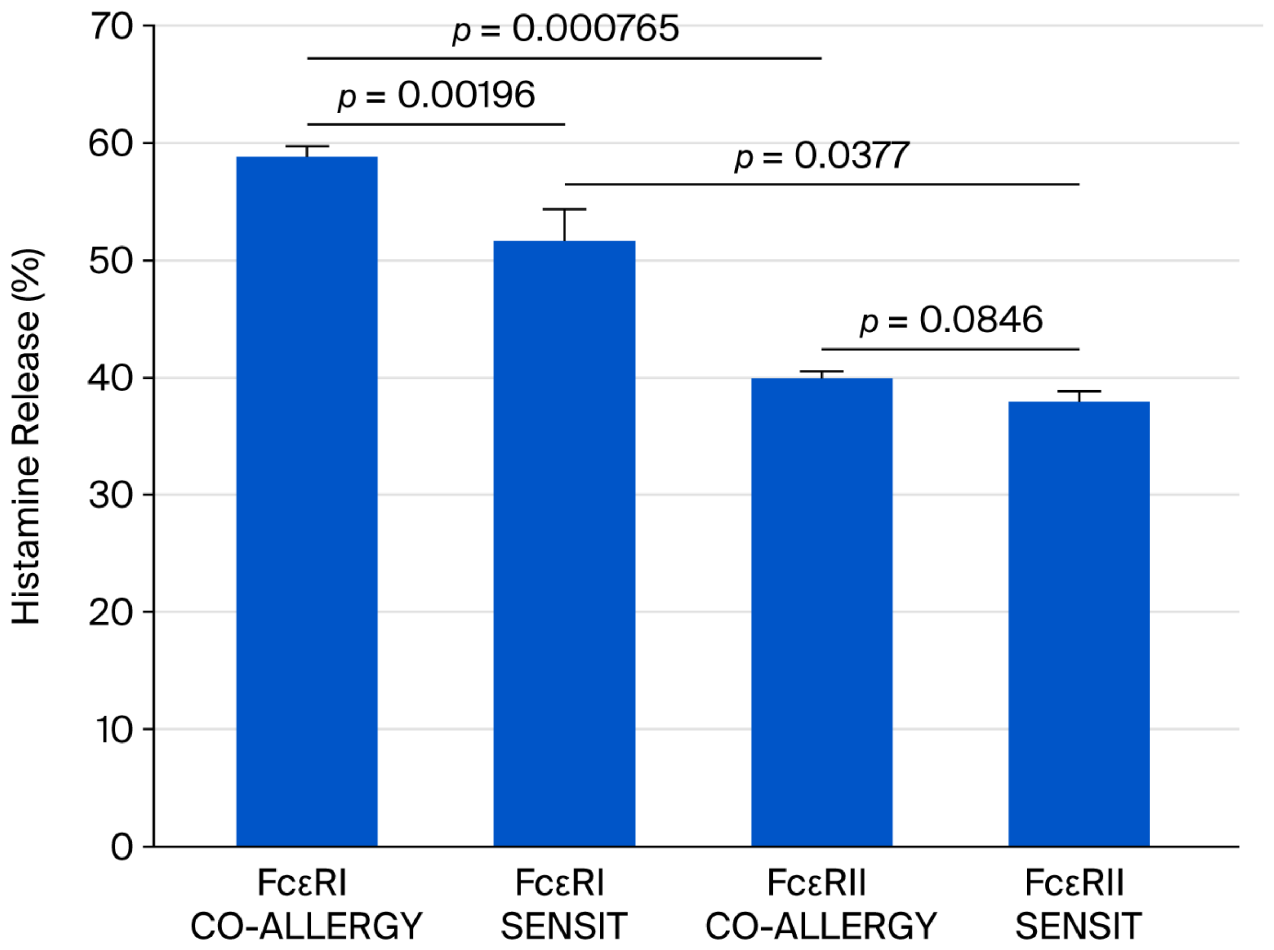
IgE receptors involved in NAT. Neutrophils release more histamine if they are stimulated with an anti-FcεRI antibody than an anti-FcεRII/CD23 antibody (5 × 10^6^ cells/300 µL). There is a greater release of histamine if neutrophils are stimulated with an anti- FcεRI antibody from patients with Co-Allergy (CO-ALLERGY) (*n* = 34) than if cells from sensitized patients are stimulated with the same antibody (SENSIT) (*n* = 16). (*p* = 0.00196). There are no significant differences if neutrophils are stimulated with an anti- FcεRII/CD23 antibody and if they are stimulated from patients with co-allergy and sensitized (*p* = 0.0846). There were differences in the histamine released by neutrophils if they were stimulated with an anti-FcεRI antibody in patients with co-allergy compared to if the cells of the same patients were stimulated with an anti-FcεRII/CD23 antibody (*p* = 0.000765), and the same situation was found in sensitive patients (*p* = 0.0377). The amount of histamine was measured based on the mean of the duplicate determinations and expressed as a percentage of histamine release by dividing by total cellular histamine after subtracting for spontaneous release of unstimulated neutrophils.

As previously described [19], neutrophils release more histamine if they are stimulated with an anti-FcεRI antibody than an anti-FcεRII/CD23 antibody, whereas there is no apparent release if the cells are stimulated with an anti-Galectin-3 antibody [19]. There is greater histamine release if neutrophils are stimulated with an anti-FcεRI antibody from allergic patients than if cells from sensitized patients are stimulated with the same antibody (*p* = 0.00196). There are no significant differences if neutrophils from allergic and sensitized patients are stimulated with an anti-FcεRII/CD23 antibody (*p* = 0.0846). There are differences between the histamine released by neutrophils if we stimulate them with an anti-FcεRI antibody in allergic patients compared to if we stimulate the cells of the same patients with an anti-FcεRII/CD23 antibody (*p* = 0.000765), and the same situation is found in sensitive patients (*p* = 0.0377) (Figure 6).

**Figure 7:**
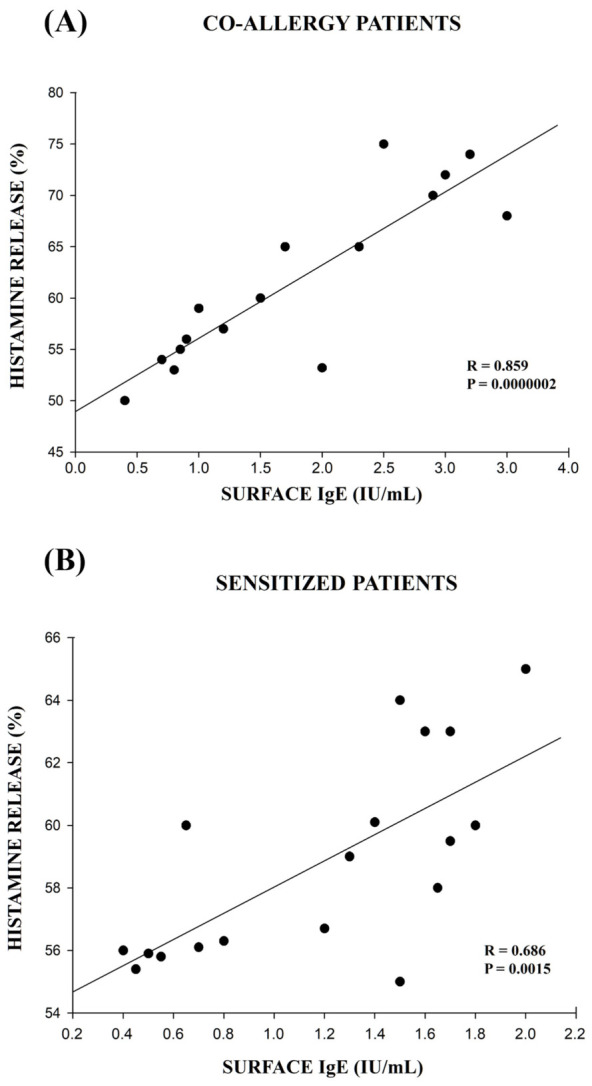
Correlation between the amount of IgE eluted from the cell surface and the amount of histamine released after specific stimulation of neutrophils both in patients who present Co-Allergy (**A**) and in sensitized patients (SENSIT) (**B**). There is a positive correlation between the amount of IgE eluted from the cell surface and the amount of histamine released after stimulation of neutrophils with the allergen, both in patients who present Co-Allergy (*n* = 34) (**A**) and in sensitized patients (SENSIT) (*n* = 16) with negative SPT and sIgE and positive ASCC (**B**). IU/mL: International Units/milliliter.

If we quantify the amount of IgE eluted from the cell surface using ELISA, there is a positive correlation with the amount of histamine released, after stimulation of neutrophils with the allergen to which the patient is sensitive, both in allergic patients (R = 0.859; *p* = 0.0000002) (Figure 7A) and in sensitized patients with negative SPT and sIgE and positive ASCC (R = 0.686; *p* = 0.0015) (Figure 7B).

## 4. Discussion

In this study, we identified a high proportion of patients with bronchial asthma presenting evidence of LAA despite negative conventional usual allergy tests. In our cohort, approximately 50% of asthmatic patients showed allergen-specific responses detectable only through ASCC, supporting the presence of localized allergic mechanisms. In addition, we demonstrate that neutrophils from these patients are capable of releasing histamine through an IgE-dependent mechanism following allergen stimulation, extending previous observations of neutrophil participation in allergic inflammation.

To our knowledge, this is the first report describing both the frequency of TLA and the functional IgE-dependent responsiveness of neutrophils in a primary asthma cohort not preselected for local allergic rhinitis (LAR). Importantly, the observed frequency should be interpreted within the context of this selected population and diagnostic strategy, which differs from previous studies evaluating progression from LAR to asthma, where lower rates have been reported [11].

These findings support the concept that localized allergic responses may remain undetected when diagnosis relies exclusively on skin prick testing or serum-specific IgE measurements, thereby expanding current understanding of asthma heterogeneity.

It is not uncommon for patients who present symptoms at one time of year and have positive SPT and sIgE to present symptoms at another time of year with negative SPT and sIgE to other allergens. The ASCC model offers a simple and reliable technique for monitoring the clinical and inflammatory changes that can be revealed by any APT. Given its safety, it can even be used several times in the same patient. It is a widely used method and provides essential information to confirm the diagnosis of sensitization to a specific allergen [16,20,21,22,23,24,25,26,27,28,29,30,31,32].

It has been shown that the ASCC model can demonstrate susceptibility to developing an allergic process with high sensitivity [33,34,35].

There are patients who present symptoms with certain SPTs and sIgE, but at the same time present symptoms that are not compatible with any SPT or sIgE. In these patients, an ASCC is performed, and previously undetected sensitizations are revealed. We call this circumstance co-allergy. We have found that in patients with bronchial asthma, approximately 52.9% presented this circumstance, i.e., LAA. To our knowledge, this is the first time this possibility has been described, which gives us the opportunity to discover new sensitizations distinct from those we had described in patients who presented symptoms with different allergens and who were not clearly defined previously. This circumstance can likely be found in other LA phenotypes. We have demonstrated here how an APT can be used to determine whether allergic individuals who already present symptoms with positive SPT and sIgE to one allergen may be sensitized to another allergen(s) but present negative SPT and sIgE to that/those allergen(s). Furthermore, as a second possibility, ASCCs are used to diagnose patients with allergy symptoms but negative SPT and sIgE (in our case, approximately 47.1%). Overall, we describe how approximately 50% of asthma cases may have TLAA. However, we assume that this frequency may be influenced by several factors, such as ethnic group, race, country, type of study, type of test used for diagnosis, etc. [36,37].

Despite the extensive and brilliant work of Rondón C’s team, they have only described the frequency of LAA in patients who already suffer from LAR, whereas we have described the total frequency of LAA (LAT) [11].

The importance of performing a basophil histamine test in these cases has been previously described [33].

We wanted to test the usefulness of the in vitro NAT, as previously described. As we can see, neutrophils are capable of releasing histamine, even in patients who only show sensitivity to the allergen studied, without presenting positive SPT and sIgE to that allergen. However, the amount of histamine released is statistically significantly lower (*p* = 0.02) than that released by neutrophils from patients who already demonstrate positive SPT and sIgE to other allergens. This could mean that when we find positive SPT and sIgE results, it may signify a further step in the overall development of the allergic process. There is no histamine release if neutrophils are not stimulated in both types of patients. Further studies are needed for a proper description and interpretation of the NAT in this type of patient.

To verify that the presence of IgE on the cell surface is essential for the NAT, we eluted this immunoglobulin from the cell surface of neutrophils. We found that if we eluted this immunoglobulin from the cell surface, the patients’ neutrophils did not release histamine, whether we stimulated the cells with an allergen to which the patient was sensitive or with an anti-IgE antibody. However, they did release histamine if we eluted IgE from the surface of neutrophils and stimulated them with an activator such as LPS, which acts as the prototypical endotoxin because it binds the CD14/TLR4/MD2 receptor complex [19,38]. We have shown that these receptors were ineffective in other cells, such as basophils [19].

The same occurs if neutrophils are stimulated with an anti-IgE antibody. No histamine is released when IgE is eluted from the cell surface in either type of patient. Neutrophils release more histamine if they are stimulated with an anti-FcεRI antibody than an anti-FcεRII/CD23 antibody, whereas there is no apparent release if the cells are stimulated with an anti-Galectin-3 antibody [19]. We found that histamine release was statistically higher (*p* = 0.00196) in allergic patients who were stimulated with an anti-FcεRI antibody than in those who were only sensitized, and we also found statistically significant differences (*p* = 0.000765) between allergic patients when we stimulated neutrophils with an anti-FcεRI antibody than when we stimulated them with an anti-FcεRII/CD23 antibody. The same situation occurred in sensitive patients (*p* = 0.0377). We did not find any differences (*p* = 0.0846) in histamine release between the two groups of patients if we stimulated neutrophils with an anti-FcεRII/CD23 antibody (Figure 6).

To our knowledge, this is the first time that these findings have been described. We present a possibility that patients who are sensitized, but who do not yet have positive SPT and IgE, may present with local allergy in proportions of 50%. We sought to demonstrate how neutrophils can play a very important role in the pathophysiology of local allergy. Thus, we have demonstrated how the NAT can be an important element for the diagnosis and the subsequent treatment of LA. Although sIgE is the fundamental and indispensable immunoglobulin for the development of the allergic reaction, IgE-expressing B cells are not initially capable of forming memory B cells (MBCs). After inhalation of the allergen, the formation of cells responsible for class switching begins, such as IgG1+ MBCs, which, together with CD4 lymphocytes, are the precursors of IgE-producing cells. IgE bound to the cell membrane represents such a low proportion of the body’s IgE that it would also lead to no detectable free sIgE in serum. Furthermore, it has been demonstrated that the production of this local IgE is independent of the production of usual IgE in circulation, necessitating a subsequent process to sensitize the different cells and organs responsible for the clinical picture. Among these, the participation of the innate immune system has been demonstrated through the phenomenon known as trained immunity (TRIM), with the participation of neutrophils. This has been the focus of attempts to modulate the allergic response that develops and determines the clinical course of these patients [39,40,41,42,43,44].

All patients with cell surface IgE had a positive ASCC, whereas no patient without cell surface IgE had a positive ASCC. There was a positive correlation between the amount of specific IgE on the cell surface and the amount of histamine released by those cells, both in allergic patients (R = 0.859, *p* = 0.0000002) and in those with only sensitization (R = 0.686, *p* = 0.0015). The strength of the correlation was lower in the latter group of patients (Figure 7A,B). To our knowledge, this is the first time that such a circumstance has been described.

Other authors and we have shown that neutrophils, traditionally associated with non-allergic asthma, may intervene through an IgE-dependent mechanism and may play a crucial role in allergic processes. As we have demonstrated here, neutrophils are capable of releasing histamine in response to stimulation with the allergen to which the patient is sensitized, both in patients with conventional allergic asthma and in those with LAA.

The discovery of local allergic symptoms within the various forms of bronchial asthma, such as LAA, is of significant importance not only for diagnosis but also for treatment. Traditional tests for diagnosing allergic symptoms, such as SPT and sIgE can miss these cases, which can lead to misdiagnosis and suboptimal treatment. Incorporating APT into the diagnostic arsenal could help more accurately identify patients with local allergic asthma.

### Study Limitations and Future Directions

Several limitations should be considered when interpreting these findings. First, this was a single-center study with a relatively limited sample size, which may influence frequency estimates and limit generalizability to broader asthma populations. Second, patient selection was based on individuals undergoing detailed allergy evaluation, potentially enriching the cohort for suspected local allergy.

Moreover, the cross-sectional design does not allow assessment of the temporal relationship between local sensitization and progression toward usual allergy. Future multicenter studies including longitudinal follow-up are needed to confirm frequency estimates, validate NAT as a diagnostic tool, and clarify the clinical relevance of IgE-dependent neutrophil activation in disease progression and therapeutic response.

## 5. Conclusions

In this study, approximately half of the evaluated asthma patients demonstrated evidence of local allergic asthma detectable only through allergen provocation testing, highlighting the potential contribution of localized allergic mechanisms in a selected clinical population. We further show that neutrophils from these patients release histamine through an IgE-dependent mechanism following allergen stimulation, supporting a functional role for neutrophils in local allergic responses.

Although these findings require confirmation in larger and multicenter cohorts, they suggest that incorporation of organ-specific provocation tests and functional cellular assays may improve identification of patients with otherwise undetected allergic asthma phenotypes. Future studies should focus on independent validation of frequency estimates and on determining the clinical and therapeutic implications of IgE-mediated neutrophil activation in asthma.

## Figures and Tables

**Table 1:** Epidemiological and clinical data of the study subjects.

	Co-Allergy	Sensitized	Health
**Subjects (n)**	34	34	15
**Race**	Caucasian	Caucasian	Caucasian
**Age (years, inclusion range)**	18–70	18–70	18–70
**Gender male/female**	20/14	18/16	8/7
**Current or ex–smokers**	0	0	0
**Representative total IgE**	≈350 UI/ML	≈270 UI/ML	≈15 UI/ML
**Skin prick test positive/negative**	16 (47.1%)/18 (52.9%)	0 (0%)/34 (100%)	0 (0%)/15 (100%)
**Specific blood IgE positive/negative**	16 (47.1%)/18 (52.9%)	0 (0%)/34 (100%)	0 (0%)/15 (100%)
**ASCC positive**	34 (100%)	16 (47.1%)	0 (0%)
**IgE surface cell specific/non-specific**	34 (100%)/0 (0%)	16 (47.1%)/18 (52.9%)	0 (0%)/15 (100%)

The data in this table are expressed as absolute numbers, percentages, or ranges (age), and have a descriptive function for each group, not a comparative one. Total IgE values are approximate representative values intended to describe the typical IgE levels observed in each group and are not presented for statistical comparison.

## Data Availability

All study data are available upon request to the authors. Clinical data and other results are not published to protect patient privacy.
